# The Correlation Between Fluid Distribution and Swelling or Subjective Symptoms of the Trunk in Lymphedema Patients: A Preliminary Observational Study

**DOI:** 10.1089/lrb.2020.0075

**Published:** 2021-06-15

**Authors:** Fumiya Hisano, Shiori Niwa, Keisuke Nakanishi, Ayana Mawaki, Kaoru Murota, Atsushi Fukuyama, Yukari Takeno, Sachiyo Watanabe, Etsuko Fujimoto, Chika Oshima

**Affiliations:** ^1^ Department of Nursing, Nagoya University Graduate School of Medicine, Nagoya, Japan.; ^2^ Department of Radiological Science, Japan Health Care College, Sapporo, Japan.; ^3^ Faculty of Nursing, Kansai Medical University, Moriguchi, Japan.

**Keywords:** breast cancer-related lymphedema, magnetic resonance imaging, manual lymph drainage

## Abstract

***Background:*** Manual lymph drainage (MLD) is one of the common treatments for breast cancer-related lymphedema (BCRL). Although the primary goal of MLD is to drain the excessive fluid accumulated in the affected upper limb and trunk to an area of the body that drains usually, the use of MLD is decided based on swelling and subjective symptoms, without assessing whether there is fluid accumulated in the affected region. The purpose of this study was to examine truncal fluid distribution in a sample of BCRL patients and investigate any correlation between such fluid distribution and swelling or subjective symptoms.

***Methods and Results:*** An observational study was conducted with 13 women who had unilateral, upper extremity BCRL. Fluid distribution was evaluated by using two magnetic resonance imaging (MRI) sequences: half-Fourier acquisition single-shot turbo spin echo and three-dimensional double-echo steady-state. The presence of swelling was determined by lymphedema therapists, and subjective symptoms were measured by using a visual analog scale. On MRI, no participants had any free water signals in the trunk. However, seven had swelling and all 13 had some kind of subjective symptoms on the affected side of their trunk.

***Conclusions:*** These results suggest that swelling and subjective symptoms do not correlate with the presence of truncal fluid. For such cases, a different approach than MLD may be needed to address truncal swelling and related subjective symptoms. Checking for the presence of fluid in the truncal region may help MLD be used more appropriately.

## Introduction

After breast cancer treatment, 8%–56% of patients suffer from breast cancer-related lymphedema (BCRL).^1^ This edema frequently results from blockage of the lymph drainage route in the axilla after mastectomy and lymphadenectomy. Axillary lymph nodes are junctions that drain lymph from ipsilateral arm, breast, and nearby areas into deep networks of lymphatic vessels. Accordingly, lymphedema often develops in the trunk and upper limb on the same side as the affected breast.^2,3^

Truncal lymphedema can cause some degree of physical and psychological sequelae (e.g., discomfort, heat, or difficulty sleeping).^4,5^ However, to date, BCRL studies mainly have focused on the upper limb rather than the trunk, as lymphedema is more apparent in the arm than in the trunk.^3^ Therefore, truncal fluid accumulation in BCRL patients remains largely unexamined.

Manual lymph drainage (MLD) is one of the most common treatments for edema in the trunk and upper limbs of BCRL patients.^6^ Although the primary goal of MLD is to drain the excessive fluid accumulated in the affected upper limb and trunk into a normally draining area, the decision to use MLD is based on the presence of swelling and subjective symptoms, without determining whether there is fluid accumulated in the region.

Recently, Niwa et al. reported that magnetic resonance imaging (MRI) showed that some BCRL patients' affected upper limbs had no fluid signals despite being swollen.^7^ This finding suggested that swelling might not correlate with the presence of fluid in the upper limb. Though not yet studied, this finding might be true for the trunk, as well. Therefore, the purpose of this study was to use MRI to examine truncal fluid distribution in BCRL patients, and to investigate the correlations between fluid distribution, and swelling or subjective symptoms.

## Materials and Methods

### Research design

This study used an observational design and was conducted from February 2019 to February 2020.

### Participants and setting

Patients with unilateral BCRL were recruited from among those referred for lymphedema care to the lymphedema outpatient unit of Nagoya University Hospital or the Japanese Red Cross Nagoya Daini Hospital.

All participants were assessed for eligibility by using the following criteria. First, they had been diagnosed with BCRL by a physician. To diagnose lymphedema, the affected arm circumference must have exceeded the contralateral arm by >1 cm. This threshold level has been demonstrated to be adequate for the diagnosis of lymphedema in Japanese BCRL patients.^8^ Second, they had received primary and adjuvant breast cancer treatment (e.g., chemotherapy or radiation therapy) that was completed at least 6 months before the study. Those receiving ongoing hormonal therapy were allowed to participate. Third, their physician had given permission for them to have the MRI examination.

### Ethical considerations

This study was approved by the Ethics Committee of Nagoya University (no. 18-138, 2018-0280-2) and the Japanese Red Cross Nagoya Daini Hospital (no. 1323), and it conforms to all conventions governing ethical conduct as stated in the Declaration of Helsinki. All participants gave their informed consent before study inclusion, and their anonymity was preserved.

### Evaluation of fluid distribution

Fluid distribution was examined by using MRI. This type of imaging is appropriate for evaluating lymphedema status, because it provides excellent contrast for visualizing soft tissues and fluid accumulation without ionizing radiation.^9,10^

Images were obtained by using a 3-Tesla MRI scanner (MAGNETOM Verio 3T; Siemens healthcare GmbH, Erlangen, Germany). Two sequences were used: half-Fourier acquisition single-shot turbo spin echo (HASTE) and three-dimensional double-echo steady-state (3D-DESS).

The 3D-DESS sequence is suitable for three-dimensional observation of fluid accumulation in lymphedematous tissue and was chosen to examine imaging differences between each participant's trunk and affected upper limb.^7^

The 3D-DESS imaging parameters were as follows: repetition time = 14.16 ms; echo time = 5.00 ms; flip angle = 28°; bandwidth = 250 Hz/pixel; field of view = 256 mm; number of slabs = 1; slices per slab = 160; and slice thickness = 1.00 mm. To reduce the physical burden on subjects, the acquisition time was shortened by using generalized auto-calibrating partial acquisition, a type of simultaneous acquisition of spatial harmonics. The parallel acquisition technique factor was set to 2. Moreover, to lessen image distortion, a two-dimensional distortion correction filter was used. The imaging range was limited to the affected side, from the midsagittal plane of the upper body. The imaging area was divided into three sections: the upper region (shoulder joint and chest), the middle region (elbow and upper abdomen), and the lower region (wrist and lower abdomen). The acquisition time was approximately 8 minutes per section, for a total of 24 minutes for one side.

HASTE is a high-speed turbo-spin echo T2-weighted sequence. This sequence was selected to provide an image of the whole trunk, as it can be used with a short breath-holding time and was suitable for observing free water, which has a long transverse relaxation time.

The HASTE imaging parameters were as follows: repetition time = 700 ms; echo time = 69 ms; flip angle = 120°; bandwidth = 391 Hz/pixel; field of view = 360 mm; number of slabs = 24; and slice thickness = 6.00 mm. The breath-holding time was 20 seconds, the fat suppression was set at *none*, and the spectral attenuated inversion recovery was set at *strong.*

The presence of signal intensity areas was determined by the consensus of three experienced observers using three-dimensional maximum intensity projection analysis of the MRI images.

### Evaluation of swelling and subjective symptoms

Truncal swelling was evaluated by the inspection and palpation of the participants by the therapists-in-charge of lymphedema patients. The lymphedema therapists classified each participant into one of two groups: those with and those without truncal swelling.

Subjective symptoms (tightness, limited arm use, heaviness, fullness, pain, and numbness) were measured by using a visual analog scale.^11^ These particular symptoms were selected from previous studies conducted to verify the effectiveness of BCRL treatment.^12^

## Results

### Sample characteristics

Table 1 shows the sample characteristics. Thirteen patients participated in this study. The mean age (±standard deviation [SD]) was 58.6 ± 9.7 years. The mean body weight (±SD) was 56.3 ± 9.6 kg. The mean lymphedema duration was 2.23 years. All participants had unilateral BCRL. Eleven participants had undergone a unilateral mastectomy, and one had undergone a bilateral mastectomy. Of the participants, 18.2% had a history of unilateral axillary lymph node dissection at Level 1, 72.7% at Level 2, and 9.1% at Level 3. Ten participants had lymphedema on their dominant limb. All of the participants were diagnosed with grade-2 lymphedema according to International Society of Lymphology (ISL; 2016) classification parameters.

**Table 1.** Sample Characteristics

	*n* (%)	Mean ± SD	Min	Max
Age (years)		58.6 ± 9.7	47	74
Weight (kg)		56.3 ± 9.6	41.9	78.2
Body mass index (kg/m^2^)		23.2	19.1	30.1
Duration of lymphedema (years)		2.23	0.1	6
Level of axially lymph node dissection				
I	2 (18.2)			
II	8 (72.7)			
III	1 (9.1)			
Unknown	2			
Lymphedema in dominant limb	10 (76.9)			
ISL classification: Grade2	13 (100)			

ISL, International Society of Lymphology; SD, standard deviation.

### Magnetic resonance imaging

**FIG. 1.**
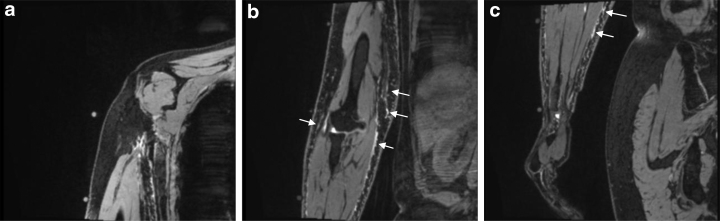
MR coronal images **(a**: upper region, **b**: middle region, **c**: lower region**)**. Hyper-intense signals were observed in their subcutaneous tissue of upper limb (*arrow*), but no signals were observed in their trunks. MR, magnetic resonance.

Eleven of the 13 participants were observed by using both the 3D-DESS and HASTE sequences, and two were observed by using only 3D-DESS. For the 3D-DESS sequence, eight participants had hyper-intense signals in the upper, middle, and lower regions of their affected upper limbs, but not in the subcutaneous area of their trunks (Fig. 1a–c). Five participants had no hyper-intense signals in either upper limb or trunk except the joint area.

**FIG. 2.**
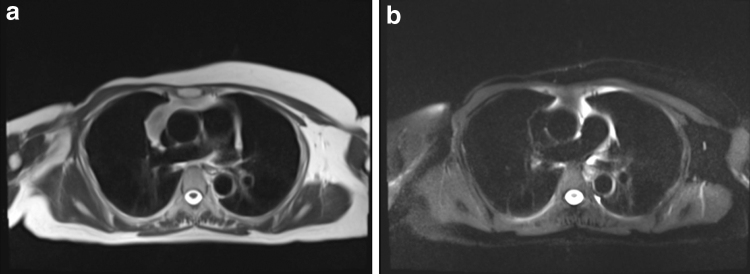
MR transverse images in the chest **(a**: none fat suppression, **b**: SPAIR, strong**)**. No hyper-intense signals were observed in their subcutaneous area. SPAIR, spectral attenuated inversion recovery.

**FIG. 3.**
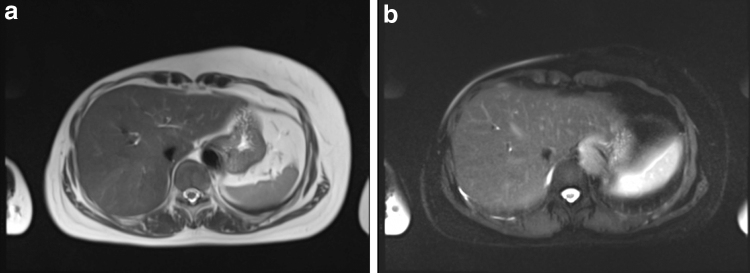
MR transverse images in the upper abdomen **(a**: none fat suppression, **b**: SPAIR, strong**)**. No hyper-intense signals were observed in their subcutaneous area.

**FIG. 4.**
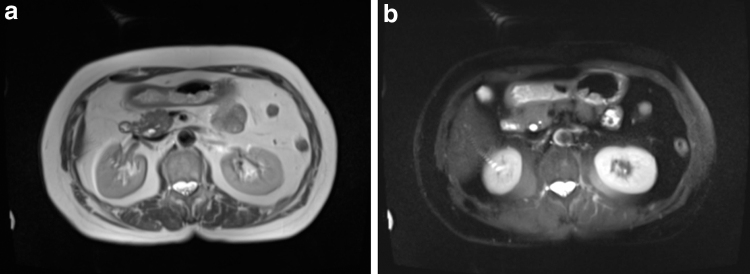
MR transverse images in the lower **(a**: none fat suppression, **b**: SPAIR, strong**)**. No hyper-intense signals were observed in their subcutaneous area.

The HASTE sequence showed that none of the participants had hyper-intense signals in the subcutaneous area, but all participants had such signals in either their stomach or spine (Figs. 2–4).

### Swelling and subjective symptoms

The lymphedema therapist determined that seven participants had truncal swelling. The number of participants with subjective symptoms is shown in Table 2. Of the 13 participants, 76.9% had tightness, 53.8% had limited arm use, 92.3% had heaviness, 69.2% had fullness, 23.1% had pain, and 15.3% had truncal numbness. All participants had some type of subjective symptoms in both the upper limb and the trunk. Table 3 shows a summary of these results.

**Table 2.** Number of Participants with Subjective Symptoms

	*n* (%) of applicable people
Tightness	10 (76.9)
Limited arm use	7 (53.8)
Heaviness	12 (92.3)
Fullness	9 (69.2)
Pain	3 (23.1)
Numbness	2 (15.3)

**Table 3.** Summary of Results

	*n* (%) in trunk	*n* (%) in upper limb
Hyperintense signals	0 (0)	8 (61.5)
Swelling	7 (53.8)	13 (100)
Subjective symptoms	13 (100)	13 (100)

## Discussion

### Fluid distribution in the trunk

In fluid-sensitive MRI sequences, hyper-intense signals indicate the presence of free water and the distinctive patterns found in the affected upper limbs of BCRL patients.^9,13^ This study was the first to use MRI to assess fluid distribution in the trunks of BCRL patients. Our results suggest that none of the participants had free water in the truncal region, and eight had free water only in their upper limbs.

According to Fumiere et al. and Tassenoy et al., as lymphedema progresses and becomes chronic, hyper-intense MRI signal images tend to lessen due to the progression of fibrillization.^10,14^ In our study, the reason for the absence of the free water is not clear, as the relationship between lymphedema's progress or chronicity and fluid distribution remains unknown. However, it has been suggested that the rate of fluid discharge in the upper limbs may differ from that of the truncal region.

### Correlation between fluid accumulation and swelling or subjective symptoms

Our MRI results suggest that none of the participants had free water in the trunk. In contrast, seven participants were assessed as having truncal swelling, and all had some subjective symptoms in the trunk. These results suggest that there is no correlation between swelling conditions or subjective symptoms and the presence of fluid in the trunk. Therefore, we posit that swelling and subjective symptoms are caused by something other than the presence of fluid. It is possible that the swelling and subjective symptoms are due to asymmetric development of muscle or fat tissue resulting from surgical wounds.

### Strengths and limitations

This study is the first to assess BCRL participants for the presence of fluid in the truncal region by using MRI. However, because the results indicated that the participants did not have fluid accumulation in the trunk, it remains unclear how fluid is distributed in the truncal region of BCRL patients. Further studies with more patients are required to confirm this study's results.

### Implications for practice

To ensure the process of draining lymph from a lymphedematous arm by using MLD is effective, truncal fluid generally is drained first. Therefore, draining fluid from the trunk is important not only for reducing patient discomfort but also for ensuring the success of lymphedema treatment for the upper limb.^2,15^ However, our results showed that BCRL patients did not have free water in the trunk despite exhibiting swelling or related subjective symptoms. Considering the absence of free water in the truncal region, MLD in this region may not be necessary for such patients. Instead, a different approach may be needed to address truncal swelling or subjective symptoms. Therefore, checking the truncal region for the presence of fluid by using MRI may allow MLD to be used more appropriately.

## Conclusions

Our findings suggest that swelling conditions or subjective symptoms in grade-2 BCRL patients do not necessarily correlate with the presence of fluid in the trunk.

## References

[1] Paskett DE, Naughton JM, McCoy PT, Case LD, Abbott MJ. The epidemiology of arm and hand swelling in premenopausal breast cancer survivors. Cancer Epidemiol Biomarkers Prev 2007; 16:775–782 17416770 10.1158/1055-9965.EPI-06-0168 PMC4771019

[2] Roberts CC, Levick JR, Stanton AW, Mortimer PS. Assessment of truncal edema following breast cancer treatment using modified Harpenden skinfold calipers. Lymphology 1995; 28:78–88 7564495

[3] Mazor M, Smoot JB, Mastick J, Mausisa G, Paul MS, Kober MK, Elboim C, Singh K, Conley YP, Mickevicius G, Field J, Hutchison H, Miaskowski C. Assessment of local tissue water in the arms and trunk of breast cancer survivors with and without upper extremity lymphoedema. Clin Physiol Funct Imaging 2019; 39:57–64 30207039 10.1111/cpf.12541 PMC6289797

[4] Ridner HS, Murphy B, Deng J, Kidd N, Galford E, Dietrich SM. Advanced pneumatic therapy in self-care of chronic lymphedema of the trunk. Lymphatic Res Biol 2010; 8:209–215 10.1089/lrb.2010.0010 PMC3008167 21190493

[5] Williams FA, Moffatt JC, Franks JP. A phenomenological study of the lived experiences of people with lymphoedema. Int J Palliat Nurs 2004; 10:279–286 15284623 10.12968/ijpn.2004.10.6.13270

[6] International Society of Lymphology. The diagnosis and treatment of peripheral lymphedema. Lymphology 2016; 49:170–184 29908550

[7] Niwa S, Mawaki A, Nakanishi K, Hisano F, Takeno Y, Fukuyama A, Kikumori T, Shimamoto K, Fujimoto E, Oshima C. Breast cancer-related lymphedema with the presence or absence of accumulation of fluid: MR findings in ISL stage 2 class. Struct Funct 2020; 18:88–94

[8] Kitamura K, Akazawa H. Multi-center survey of breast cancer related arm lymphedema and future issues. J Jpn Coll Angiol 2010; 50:715–720

[9] Tassenoy A, Mey DJ, Ridder DF, Schuerbeeck VP, Vanderhasselt T, Lamote J, Lievens P. Postmastectomy lymphoedema: Different patterns of fluid distribution visualised by ultrasound imaging compared with Magnetic Resonance Imaging. Physiotherapy 2011; 97:234–243 21820542 10.1016/j.physio.2010.08.003

[10] Tassenoy A, Strijcker DD, Adriaenssens N, Lievens P. The use of noninvasive imaging techniques in the assessment of secondary lymphedema tissue changes as part of staging lymphedema. Lymphatic Res Biol 2016; 14:127–133 10.1089/lrb.2016.0011 27631582

[11] Gift AG. Visual analogue scales: Measurement of subjective phenomena. Nurs Res 1989; 38:286–288 2678015

[12] Nakanishi K, Mawaki A, Oshima C, Takeno Y, Kurono F, Taniho Y, Murotani K, Kikumori T, Fujimoto E. Nighttime bandaging to reduce lymphedema swelling: A clinical pre–post study. SAGE Open Nurs 2017; 3:1–8

[13] Gardner CG, Nickerson PJ, Watts R, Nelson L, Dittus LK, O'Brien JP. Quantitative and morphologic change associated with breast cancer-related lymphedema. Comparison of 3.0T MRI to external measures. Lymphatic Res Biol 2014; 12:95–102 10.1089/lrb.2013.0026 PMC4062115 24654879

[14] Fumiere E, Leduc O, Fourcade S, Becker C, Garbar C, Demeure R, Wilputte F, Leduc A, Delcour C. MR imaging, proton MR spectroscopy, ultrasonographic, histologic findings in patients with chronic lymphedema. Lymphology 2007; 40:157–162 18365529

[15] Thiadens RJS, Stewart JP, Stout LN. 100 Questions & Answers About Lymphedema. USA: Jones & Bartlett Learning

